# Human NK Cells Differ More in Their KIR2DL1-Dependent Thresholds for HLA-Cw6-Mediated Inhibition than in Their Maximal Killing Capacity

**DOI:** 10.1371/journal.pone.0024927

**Published:** 2011-09-19

**Authors:** Catarina R. Almeida, Amit Ashkenazi, Gitit Shahaf, Deborah Kaplan, Daniel M. Davis, Ramit Mehr

**Affiliations:** 1 Division of Cell and Molecular Biology, Imperial College London, London, United Kingdom; 2 The Mina and Everard Goodman Faculty of Life Sciences, Bar-Ilan University, Ramat-Gan, Israel; Centre de Recherche Public de la Santé (CRP-Santé), Luxembourg

## Abstract

In this study we have addressed the question of how activation and inhibition of human NK cells is regulated by the expression level of MHC class I protein on target cells. Using target cell transfectants sorted to stably express different levels of the MHC class I protein HLA-Cw6, we show that induction of degranulation and that of IFN-γ secretion are not correlated. In contrast, the inhibition of these two processes by MHC class-I occurs at the same level of class I MHC protein. Primary human NK cell clones were found to differ in the amount of target MHC class I protein required for their inhibition, rather than in their maximum killing capacity. Importantly, we show that KIR2DL1 expression determines the thresholds (in terms of MHC I protein levels) required for NK cell inhibition, while the expression of other receptors such as LIR1 is less important. Furthermore, using mathematical models to explore the dynamics of target cell killing, we found that the observed delay in target cell killing is exhibited by a model in which NK cells require some activation or priming, such that each cell can lyse a target cell only after being activated by a first encounter with the same or a different target cell, but not by models which lack this feature.

## Introduction

Natural Killer (NK) cells are lymphocytes capable of cytotoxicity and cytokine secretion, which interact with other cells and have an important role in certain anti-viral and anti-tumor immune responses. Their response is determined by the integration of activating and inhibitory signals and one important unknown is how these signals are integrated. We have previously shown that there is a clear threshold in the amount of target cell surface Human Leukocyte Antigen (HLA)-C protein required for inhibition of NK cell cytotoxicity [1]. In a separate study, small changes in expression of MHC class I expression, modulated by treating tumor cells with a combination of IFN-γ (to increase MHC-I expression) and β_2_m-siRNA (to target β_2_m mRNA and consequently inhibit MHC-I surface expression), were again found to change susceptibility of NK cells [2]. Furthermore, expression of the activating ligand MICA changes the thresholds for inhibition of NK cell cytotoxicity mediated by the target cell MHC-I levels [3]. A naturally occurring mechanism regulating expression levels of different alleles of the MHC class I protein HLA-C involves microRNAs. More specifically, miR-148a regulates HLA-C expression by binding to the varied 3′ untranslated region (UTR) of *HLA-C*, whose variants associate with control of HIV [4]. Thus it is important to elucidate the effects of MHC expression levels on NK cell inhibition.

Both the identity and quantity of inhibitory receptors on NK cells determine NK cell responsiveness [5], [6], [7], [8]. It has been qualitatively shown that murine NK cells with a low level of inhibitory receptors require a higher level of inhibitory ligands to achieve an inhibition of lysis similar to that of cells expressing a high level of receptors [9], [10]. The reason for this may be that the probability of an inhibitory receptor finding its ligand depends on the number of ligands on the target cell (and vice-versa), as shown in our recent study of the dynamics of the NK cell immunological synapse [11]. Similarly, murine NK cells expressing low amounts of Ly49 inhibitory receptors required higher amounts of purified MHC class I protein to inhibit IFN-γ production, than cells expressing high amounts of inhibitory receptors [12]. It has also been recently shown that the threshold for inhibition of target cell lysis by Ly49-expressing NK cells is quite low, with inhibition occurring even for target cells with only 20% of the MHC-I levels expressed by homozygous, normal, target cells. MHC-hemizygous target cells (with only one copy of the gene for an Ly49A ligand) had the same effect in inhibiting lysis by NK cells as homozygous target cells. Additionally, target cells from mice expressing haplotypes with much lower levels of Ly49A-tetramer binding than those of H-2^d^ (e.g. H-2^k^) were able to exert inhibition of Ly49A+ NK cells to a similar level than cells expressing H-2^d^ [13], [14]. In humans, studies have shown that inhibitory receptor expression determines NK cell responsiveness [5], [7], [15], and that Killer cell Immunoglobulin-like Receptor (KIR) expression regulates inhibition of NK lysis by target cells expressing low amounts of MHC class I protein, while target cells expressing high amounts of HLA inhibit NK cell cytotoxicity independently of KIR expression [16]. However, it remains to be clarified what precisely determines the differences in the MHC-I mediated thresholds of inhibition of lysis by human NK cells.

During development, NK cells may downregulate the self-MHC-I binding inhibitory receptors; this may be one of the mechanisms of activation threshold adjustment, in order to better sense changes in MHC expression levels in host cells [17]. Elucidating the dependence of the NK cell activation on the numbers of inhibitory receptors and ligands should provide a clearer understanding of NK cell function and its regulation. Here, we studied the activation/inhibition thresholds (defined in terms of the number of MHC ligands on the target cell) of different human peripheral blood NK cell clones using both experimental and theoretical methods. Functionally, the extent of inhibition of cytotoxicity was correlated with inhibition of cytokine secretion. Using mathematical modelling of target cell killing by various NK cell clones, we found that NK cell clones differ mostly in the activation threshold and less in their maximum killing capacity, and that HLA-Cw6-mediated inhibition thresholds were determined mainly by KIR2DL1 expression. In addition, we constructed mathematical models of NK cell activation and target cell killing and compared the kinetics predicted by these models to the experimental data on the kinetics of target cell killing. The results strongly suggest that NK cell cytotoxicity requires an initial activation or priming, such that each cell can lyse a target cell only after being activated by a first encounter with the same or a different target cell.

## Materials and Methods

### Cells

721.221 (hereon referred to as 221 cells) is an EBV-transformed human B lymphoblastoid cell line that does not express endogenous HLA-A, HLA-B and HLA-C [18]. 221 cells have been previously transfected to express different amounts of GFP-tagged HLA-Cw6 protein, with target cells 6.1 to 6.7 expressing progressively increasing amounts of MHC class I (6.1 expressing an average of 1.5×10^4^ MHC class I molecules, 6.2 expressing 2.5×10^4^ molecules, 6.3 expressing 4.5×10^4^, 6.4 expressing 7.3×10^4^, 6.5 expressing 7.5×10^4^, 6.6 expressing 8.5×10^4^ and 6.7 expressing 1.3×10^5^ surface MHC class I molecules – Table 1) [1]. Number of surface molecules expressed per cell was determined as previously with Quantum Simply Cellular beads (Bangs Laboratories) [1]. Cell lines were cultured in RPMI 1640 medium supplemented with 10% heat inactivated foetal bovine serum, 2 mM L-glutamine, 50 U/ml penicillin-streptomycin, 1x non-essential amino acids, 1 mM sodium pyruvate and 0.5 mM β-mercaptoethanol (all from Invitrogen) (from hereon called RPMI plus supplements). The 221 HLA-Cw6-GFP transfectants were supplemented with 1.6 mg/ml geneticin (Gibco). Human peripheral blood NK cells from anonymous donors were generated by magnetic sorting peripheral blood mononuclear cells, according to manufacturer's instructions (StemCell Technologies). NK cell clones were generated as previously described [1]. NK cells were grown in the presence of 100 or 200 U/ml human recombinant IL-2 (Roche or National Cancer Institute, Fisher, respectively). The purity and phenotype of the human NK clones were determined by staining for CD3 and CD56.

**Table 1:** Different target cell transfectants express varied levels of the MHC class I protein HLA-Cw6.

	Number surface MHC class I molecules	
Target cell clone	Average	SEM
6.1	1.5×10^4^	9.6×10^2^
6.2	2.5×10^4^	7.8×10^3^
6.3	4.5×10^4^	4.3×10^3^
6.4	7.3×10^4^	7.4×10^3^
6.5	7.5×10^4^	8.7×10^3^
6.6	8.5×10^4^	1.1×10^4^
6.7	1.3×10^5^	2.0×10^4^

Average number of surface MHC class I molecules and standard error of the mean (SEM) are shown for each target cell clone.

### Antibodies

The following antibodies were used at 10 µg/ml: anti-NKG2A (131411, R&D systems), anti-NKp46 (195314, R&D systems), anti-CD94 (HP-3D9, BD Pharmingen), anti-KIR2DL/S1 (EB6, Serotec). Anti-LIR1 (HPF1, gift from M. Lopez-Botet) was used at 1∶100 for flow cytometry. PE-Cy5-labelled anti-CD56 (B159, BD Pharmingen) and PE-Cy5-labelled anti-CD3 (UCHT1, BD Pharmingen) were used at 1∶25 for flow cytometry. PE-labelled anti-CD107a (H4A3, BD Pharmingen) was used at 7 µl per test. APC-labelled anti-IFN-γ (B27, BD Pharmingen) was used at 2 µg/ml for intracellular staining. For ELISA, anti-IFN-γ (NIB42, BD Pharmingen) was used at 2 µg/ml, biotin-labelled anti-IFN-γ (4S.B3, BD Pharmingen) was used at 1 µg/ml and HRP-conjugated streptavidin (BD Pharmingen) was used at 1∶1000. Adequate isotype-matched controls were all purchased from BD Pharmingen. The secondary antibody Alexa 488-conjugated goat anti-mouse IgG (Invitrogen) was used at 4 µg/ml.

### Cell surface staining for flow cytometry

10^5^ cells were incubated for 30 min at 4°C with the appropriate antibody diluted in PBS 1x/1% BSA/0.01% sodium azide. After three washes with PBS 1x/1% BSA/0.01% sodium azide, cells were incubated for 30 min at 4°C with the secondary antibody. The cells were washed twice and analysed by flow cytometry. Isotype matched antibodies were used as controls. Data was analysed with CellQuest (Becton Dickinson) or FlowJo software.

### Cytotoxicity assays

NK cells cytotoxicity against different target cells was assessed in ^35^S-Met release assays, as described previously [19]. Spontaneous release of ^35^S was less than 25% of the maximum release. For each NK cell clone, to determine the amount of MHC class I required to elicit half the maximum lysis (EC50), a logistic curve was fitted to the plot with the lysis elicited by different target cells as a function of target cell surface HLA-Cw6 and the value of EC50 was determined with Origin.

### Degranulation assay and IFN-γ intracellular staining

75,000 NK cells and 225,000 target cells were co-incubated for 5 hr at 37°C/5% CO_2_, in 100 µl of RPMI plus supplements in V-bottom 96-well plates in the presence of anti-CD107a antibody and 10 µg/ml Brefeldin A (Sigma). As a negative control, NK cells were incubated in the presence of medium only. 100 µl of PBS/5 mM EDTA/0.5% BSA was added to separate the conjugates and plates were centrifuged at 400 *g* for 3 min at 4°C. Cells were incubated in 150 µl of PBS/5 mM EDTA/0.5% BSA for at least 30 min on ice. Plates were centrifuged again and the cells were fixed by adding 200 µl of Cytofix/Cytoperm™ (BD Biosciences) and incubating for 20 min at 4°C. Cells were washed twice with 200 µl of PBS/5 mM EDTA/0.5% BSA/0.1% Tween 20. Cells were stained with an APC-conjugated anti-IFNγ mAb in Perm/Wash/1% BSA for 30 min at 4°C. Cells were washed a further two times and analysed by flow cytometry.

### Measuring IFN-γ secretion by ELISA

10^6^ NK cells were incubated with 10^6^ irradiated target cells (6,000rad) in 200 µl DMEM plus supplements in flat-bottom 96-well plates for 72 hr at 37°C/5% CO_2_. The supernatant was collected after centrifuging the plates for 10 min at 200 *g* at room temperature. ELISA plates (Maxisorp, NUNC) were incubated overnight at 4°C with 50 µl/well of anti-IFN-γ capture antibody in binding solution (0.1 M Na_2_HPO_4_, pH 9.0). Plates were washed three times with 200 µl/well of PBS/0.05% Tween 20 and blocked with 200 µl/well of blocking buffer (PBS/3%BSA) for 1–2 hrs at 37°C. After three washes with PBS/0.05% Tween 20, 100 µl/well of samples were plated in triplicates. Plates were incubated 1–2 hrs at 37°C and washed a further five times. Plates were incubated for 1 hr at 37°C with 100 µl/well of biotinylated anti-IFN-γ detection antibody in blocking buffer/0.05% Tween 20. Plates were washed six times with PBS/0.05% Tween 20 and incubated for 30 min at 37°C with 100 µl/well of streptavidin-HRP in blocking buffer/0.05% Tween 20. Plates were washed six times and 100 µl/well TMB ELISA substrate (Sigma) was added. Plates were read on an ELISA plate reader (Multiskan MCC/340, Titertek) at 620 nm. As negative controls, wells without biotinylated mAb, without streptavidin-HRP or without standards/samples were also assayed.

### Modelling the dependence of activation thresholds on MHC class I and KIR expression levels

To model the dependence of the activation/inhibition threshold on MHC class I and KIR expression levels, we assumed that the target cell killing probability per encounter, *κ*, depends on the product of MHC and KIR numbers (MHC*KIR), such that higher numbers of either will lead to higher inhibition. This assumption is based on the findings that cells with lower level of inhibitory receptors require more inhibitory ligands to achieve the same level of inhibition [9], [10], [12], as the probability of an inhibitory receptor finding its ligand depends on the number of ligands on the target cell (and vice-versa) [11]. We used a sigmoid threshold function (Equation 1), where *S* denotes the threshold of the NK cell clone in the same experiment, in units of (molecule numbers)^2^ as it is given in terms of the MHC*KIR product. The parameter *κ_max_* is the maximum killing capacity of each cell in the clone per encounter, and *n* is the exponent of the sigmoid function.
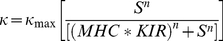
(1)


This function approaches *κ_max_* for MHC*KIR <<S (insufficient inhibition), and for MHC*KIR >>S it approaches zero (complete inhibition). Around MHC*KIR  = S, threshold sharpness is determined by the exponent *n*. We fitted this function to the data on NK cell killing of target cells with various MHC levels, in order to see whether clones differ in their activation threshold, maximal killing capacity, or both.

### Fitting the threshold function to the experimental data

Model simulation and fitting to our experimental data was done using the Berkeley Madonna© and Matlab© softwares. Equation (1) was fitted to the data on target cell killing vs. MHC level using the least-squares fitting function of Matlab. In each fitting procedure, the parameter space was scanned in small intervals, and each set of values was used as an initial guess for the software's fitting algorithm. KIR and MHC-I molecule numbers expressed by NK and target cells were measured as described [1]. Values of the threshold parameter (*S*) were varied according to the ranges of KIR and MHC-I molecule numbers. The maximum killing probability (*κ_max_*) values were varied as well, within the ranges of maximal killing (of MHC^−^ target cells) exhibited by the NK cell clones. Values of the response sensitivity exponent (*n*) were varied between 1 and 5. Due to the high variability between clones, we saw no point in obtaining confidence limits for each parameter value for each clone, as only the overall variability between clones was of interest to us.

### Modelling NK cell activation dynamics

#### Simple Model

**Figure 1:**
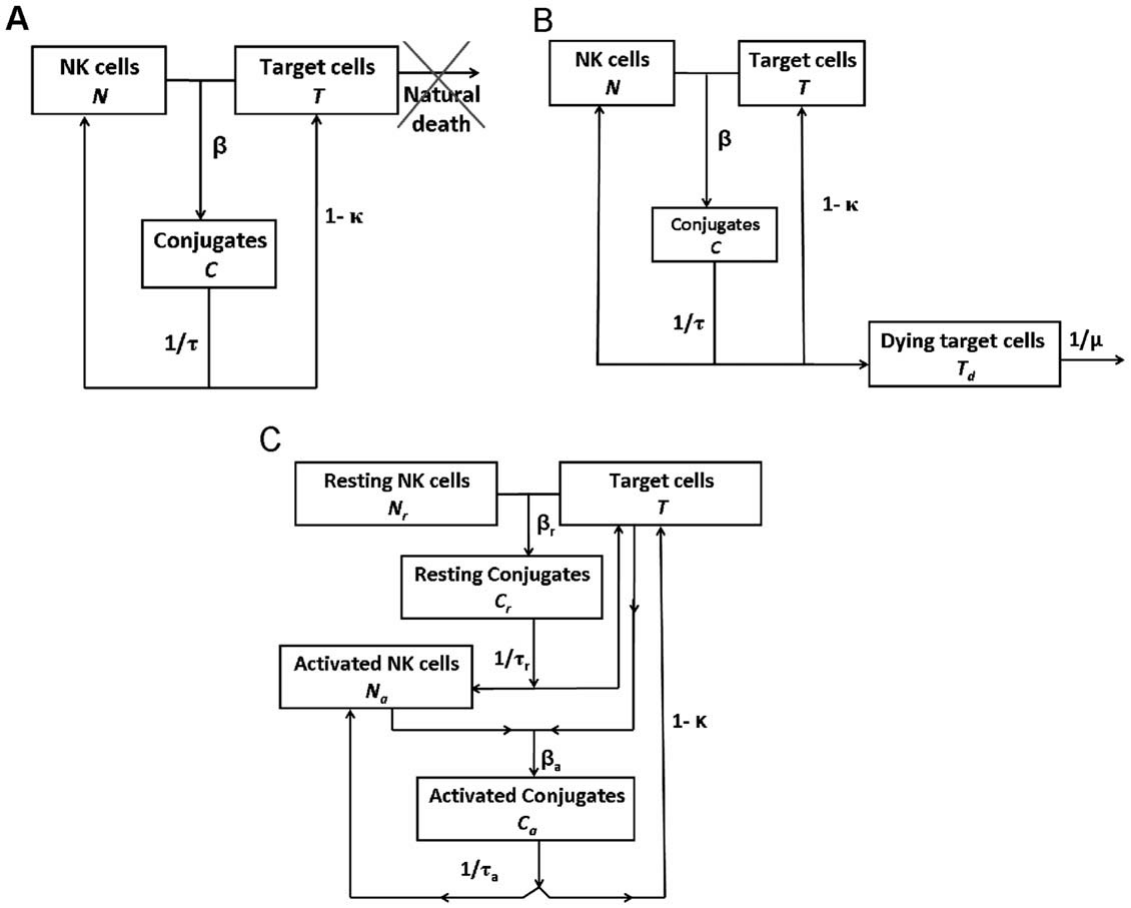
Modelling NK cell activation dynamics. **A** ) The Basic model. The variables represented by this model are: *N* – the number of free NK cells; *T* – the number of free target cells; and *C* – the number of NK-target cell conjugates. The Parameters that govern the behaviour of these populations are: *β* - the conjugation rate (cells*min)^−1^; *τ -* the conjugate lifetime (min), such that conjugates will break up at rate 1/*τ* (min)^−1^; and *κ -* the death rate of post-encounter target cells, such that (*1-κ*) is the fraction of living targets returning to the free target population. **B** ) Model with a delay in cell death. Here, *T_d_* is the number of dying target cells; they die at rate *µ* ((min)^−1^), such that *1/µ* is the dying target cell lifetime (min). **C** ) Model with a delay in cell activation. The model version shown here does not include target cell death delay. The variables here are: *N_r_* – the number of free resting NK cells; *N_a_* – the number of free activated NK cells; *T* – the number of free target cells; *C_r_* –the number of cell conjugates of target and resting NK cells; and *C_a_* – the number of cell conjugates of target and activated NK cells. Parameters are the same as in the basic model, except for different conjugation and dissociation rates for resting and activated NK cells: *β_r_* and *τ_r_* for resting and *β_a_* and *τ_a_* for activated NK cells, respectively.

In the simple model for conjugation and dissociation of target and NK cells, (Figure 1A), the number of free NK cells is denoted by *N*, the number of free target cells by *T*, and the number of NK-target cell conjugates by *C*. The following differential equations (2–4) describe the changes of these populations with time.
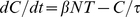
(2)

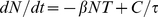
(3)

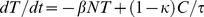
(4)


In these equations, the conjugation rate is denoted by *β* (cells*min)^−1^, such that the number of conjugates formed in every time step is *βNT*, proportional to both the number of free NK cells and the number of free target cells. Conjugate lifetime is denoted by *τ* (min), such that conjugates dissociate at rate 1/*τ* (min)^−1^. NK cell dynamics are thus the inverse of conjugate dynamics (equation 3), where *–βNT* describes NK cells moving to the conjugates population, and *C/τ* is the number of NK cells becoming free each time step due to conjugate release. The parameter *κ* denotes the death rate of post-encounter target cells, such that *(1-κ)/τ* living targets return to the free target population each minute.

#### Model with delay in target cell death

In the second model version, the dying target cells are a separate population (Figure 1B). Here the number of dying target cells is denoted by *T_d_*, such that *κC/τ* target cells destined to die after each encounter join the *T_d_* population per minute (equation 5). The *T_d_* subset death rate is denoted by *µ* ((min)^−1^).
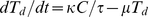
(5)


#### Model with delay in NK cell activation

To model a delay in NK cell activation (Figure 1C), we divided the NK cell population into resting and activated subsets, where resting NK cells do not kill targets. However, after their first encounter with target cells they become activated and capable of killing (equations 6–10). The numbers of free resting and activated NK cells are denoted by *N_r_* and *N_a_*, respectively, and those of target-resting NK cell and target-activated NK cell conjugates by *C_r_* and *C_a_*, respectively. Parameters are the same as in the basic model, except for different conjugation and dissociation rates for resting and activated NK cells: *β_r_* and *τ_r_* for resting and *β_a_* and *τ_a_* for activated NK cells, respectively.
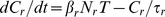
(6)

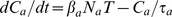
(7)

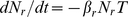
(8)

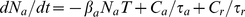
(9)

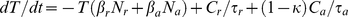
(10)


We also tried a model combining both death and activation delays (not shown), but its behaviour did not differ much from that of the model with activation delay alone.

#### Parameter values for model exploration

The data on target cell killing vs. time were not sufficient to perform quantitative fitting (there was only one data point per MHC level per time point), so we looked only for qualitative resemblance to the data.

Conjugation (*β*) and dissociation (*τ*) rates were obtained by fitting the model to the experimental data and scaling it using data from the literature, as follows. Since *κ* is negligible in the time frame of the conjugation experiments (30 minutes), the best fit of these data to the basic model with *κ* = 0 (Figure S1A) gave: *β* = 3.56×10^−6^ (cells*minutes)^−1^ and *τ* = 16.75 (minutes). Note that we do not assume there is no killing of target cells by NK cells in the first 30 minutes, but rather that the target cells that are killed would not have completed their death processes and hence would still be counted as alive in this time frame. Similar parameter values were obtained by fitting our model to another, published conjugation data set [20] (data not shown).

The lysis experiments were performed with an effector-target ratio of 10∶1, while the conjugation experiments were performed with a 1∶1 effector-target ratio. In order to scale the conjugation rate to the E:T = 10∶1 experiments, assuming that the effector-target ratio only affects conjugation, but not dissociation, we used a published data set that presents the % lysis of 221 target cells by untreated NK cells for different effector-target cell ratios [21]. The scaling factor received was 6.3, which gave *β* = 2.26×10^−5^ (cells*min)^−1^ for the experiments with a 10∶1 effector:target cell ratio.

To verify that the model behaves as expected, we ran the model with the above values for *β* and *τ*, without killing (*κ* = 0), and observed that we reach a steady state for the conjugated and the dissociated cells (Figure S1B). If we raise *κ* to 0.05, the fractions of target cells decrease with time (Figure S1C), as expected. The next step was to see how the value of *κ* affects the lysed target cell fraction after 5 hours, as in the experiments (Figure S1D): the higher the killing probability per encounter, the higher the lysed cell fraction, and the dependence is logarithmic, because killing slowly approaches 100% for high values of *κ*. Similar graphs were generated for the more advanced versions of the model (not shown).

## Results

### Cytotoxicity and IFN-γ secretion: independent activation, correlated inhibition

**Figure 2:**
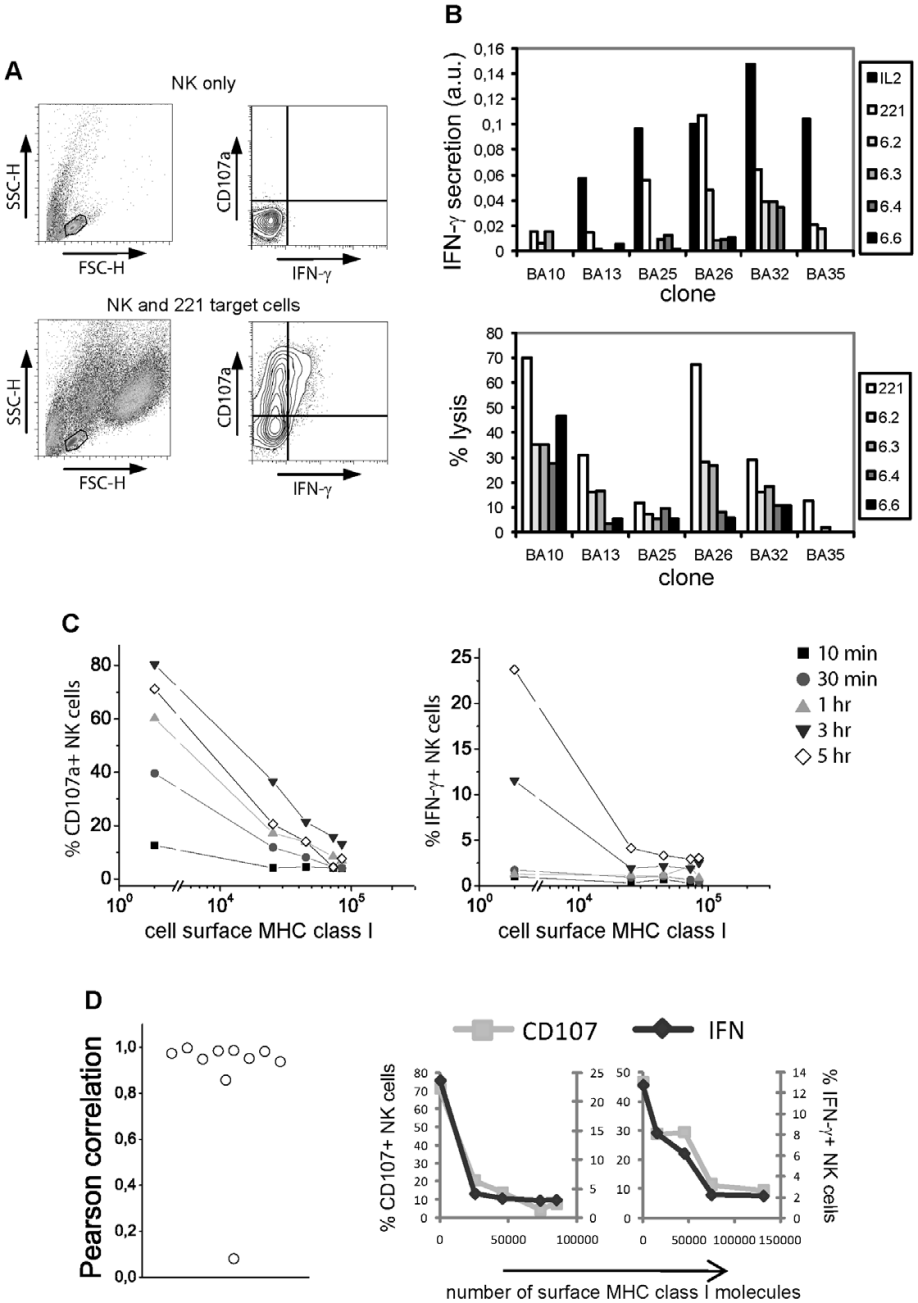
CD107a degranulation and IFN-γ production by different human NK cell clones. **A**) Induction of CD107a and IFN-γ are not correlated. NK cells were co-incubated with (bottom) or without (top) 221 target cells and analysed by flow cytometry. NK cells were selected on the basis of size (left; FSC-H stands for Forward Scatter and correlates with the cell volume while SSC-H stands for Side Scatter and correlates with the granularity of the cell; NK cells are the smaller cells, while the larger events correspond to target cells) and analysed for CD107a degranulation and IFN-γ intracellular staining (right). **B**) Top: Amount of IFN-γ, determined by ELISA, secreted by different NK cell clones incubated for 72 hr with 100 U/ml IL-2, or in the presence of irradiated target cells (221 cells expressing no MHC class I, or target cell clones 6.2, 6.3, 6.4 or 6.6, expressing an increasing number of surface MHC class I molecules per cell – Table 1). Bottom: Percent of lysed target cells by NK cell clones as determined by 5 hr radioactive release assays (bottom), using the same target cell clones. **C**) CD107a degranulation (left) and IFN-γ production (right) by NK cells incubated with different target cells for different times. Data shown was obtained with an NK cell line, and are representative of data acquired with two NK clones and one polyclonal NK cell line. **D**) Correlation between inhibition of NK cell CD107a degranulation and IFN-γ production. NK cell clones were incubated with 221 cells or cells expressing different MHC-I levels for 5 hr and analyzed for CD107a degranulation and intracellular IFN-γ production (right, two clones are shown). Left: Pearson correlation coefficients between the percent of CD107a+ and that of IFN-γ+ NK cells upon co-incubation with target cells expressing different levels of MHC-I protein, calculated with SPSS v14 for Windows. Each circle represents one NK cell clone.

We have previously shown that inhibition of NK cell cytotoxicity correlates with MHC class I protein expression by target cells [1]. To test whether other NK cell functions such as IFN-γ secretion are affected by the activation/inhibition threshold, a panel of target cells expressing varied amounts of HLA-Cw6 was used. For this purpose, 221 cells had previously been transfected to stably express different levels of GFP-tagged HLA-Cw6, named from 6.1 to 6.7 for increasing expression of MHC-I protein (Table 1 and [1]). Peripheral blood human NK cells incubated with the different target cells were stained for both CD107a degranulation and intracellular IFN-γ. It should be noted that, though CD107a release has been suggested to reflect the cytotoxic capability of CD8+ T cells and NK cells [22], [23], degranulation and cytotoxicity are not always correlated in NK cell clones (Figure S2). CD107a^+^ NK cells did not always produce IFN-γ after a 5 hr co-incubation with target cells, while all IFN-γ^+^ NK cells were CD107a^+^ (Figure 2A). Also, while all NK cell clones were stimulated with PMA/ionomycin to produce IFN-γ, not all of them secreted IFN-γ upon stimulation with 221 target cells (data not shown). Similarly, production of IFN-γ in the presence of 221 target cells was not always detected by ELISA in clones that were able to lyse the same targets, and that could secrete cytokines upon stimulation with IL-2 (Figure 2B). As expected, CD107a degranulation occurred in a shorter time frame than cytokine production (Figure 2C). These data indicate that NK cells have different requirements for induction of cytotoxicity and cytokine secretion, as also reported by others [24]. Importantly, in contrast to the different activation requirements, we found that in human NK cells, inhibition of IFN-γ production by different amounts of MHC-I protein correlated with inhibition of lytic granules release (Figure 2D).

### Expression of KIR2DL1 determines the amount of target HLA-Cw6 required for NK cell inhibition

**Figure 3:**
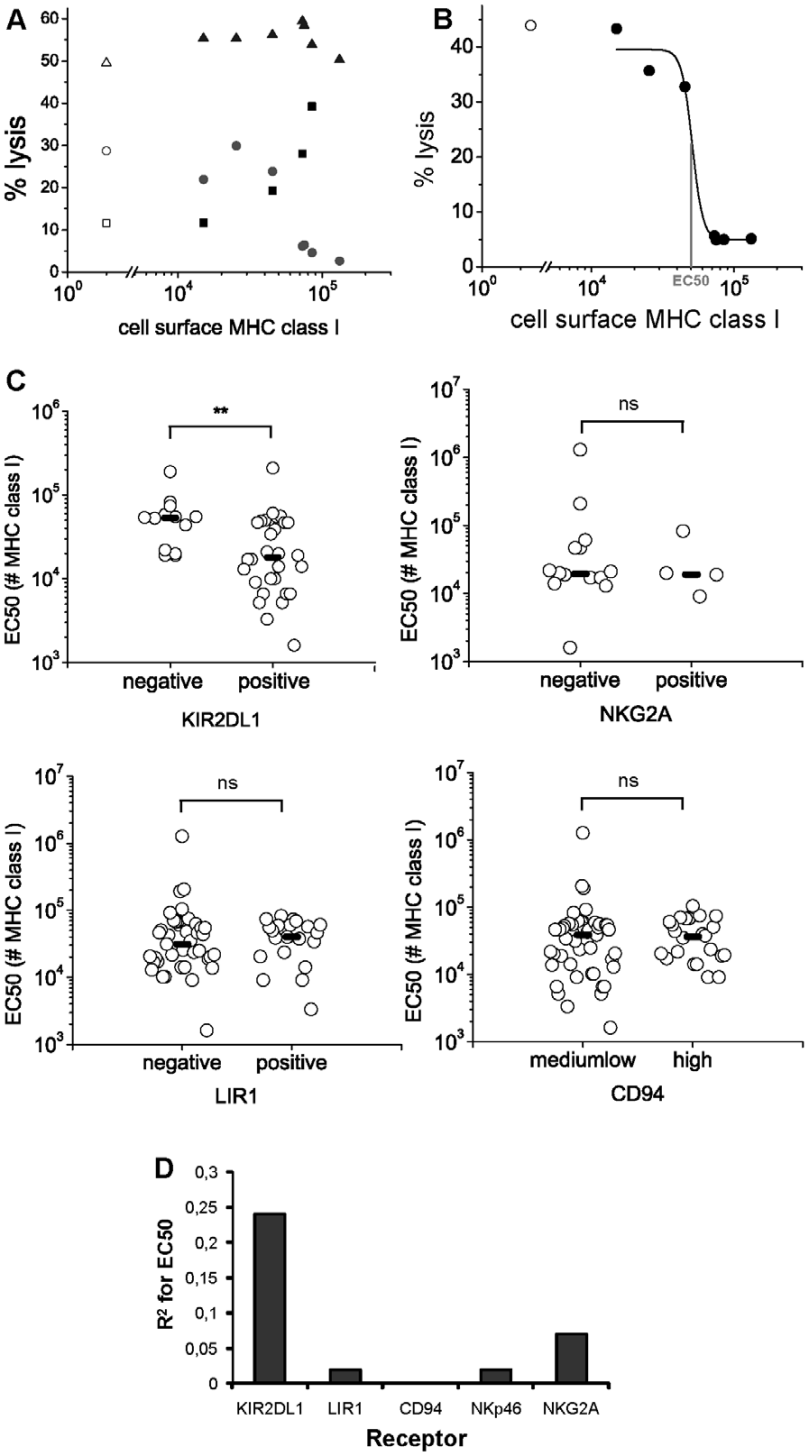
The threshold in MHC-I mediated NK cell inhibition depends on the expression level of KIR2DL1. **A**) Lysis of different target cells by three NK cell clones, as determined by radioactive release assays, plotted against target MHC-I cell surface expression. Open symbols represent lysis of untransfected 221 cells. Circles show a clone inhibited by MHC; triangles show a clone not inhibited by MHC; and squares show a clone whose activity increased as function of target MHC level. **B**) Lysis of target cells by one NK cell clone as a function of target MHC-I cell surface expression. A sigmoid curve was fitted to the data (using the Origin software) and the amount of MHC-I required to inhibit lysis to half its maximum, the EC50, was determined. **C**) Where inhibition of lysis was observed, EC50 was determined for different clones phenotyped for KIR2DL1, NKG2A, LIR1 and CD94 expression. Each clone may express one or more receptors. Medians are indicated and statistical significance of differences was determined with the non-parametric Mann-Whitney test. **P<0.005; ns, not significant. **D**) Correlation between EC50 and receptor expression level, obtained by performing a regression analysis to evaluate the contribution of each receptor – or combination of receptors – to the EC50 data. R^2^ is the correlation measure, and the higher it is, the higher the correlation. More details are given in Table S1.

Consistent with previous results, there was a sharp threshold in the amount of target cell MHC-I protein required to inhibit most NK cell clones. Interestingly, the amount of MHC-I required to inhibit cytotoxicity varied greatly among different human NK cell clones. A few clones showed no inhibition, or even activation by target cell MHC-I, likely due to expression of the activating receptor KIR2DS1 (examples are shown in Figure 3A). These different responses could be found among different clones from the same NK cell donor.

In order to find which, if any, NK cell receptors determine this threshold, expression levels of the inhibitory receptors KIR2DL1, LIR1, NKG2A and CD94 were tested (Figure 3 and Figure S3). KIR2DL1 can recognize HLA-Cw6, amongst other alleles. LIR1 can recognize HLA-A, B and C alleles, including HLA-Cw6 [25]. CD94 forms heterodimers with NKG2A and NKG2C, thus forming inhibitory and activating receptors capable of recognizing HLA-E [26], [27], [28]. NKG2A expression was variable amongst different NK cell clones, consistent with previous reports [29]. Expression of each of these receptors in different clones was then correlated with the value of EC50, i.e. the amount of MHC-I protein required for eliciting half maximum lysis (Figure 3). Importantly, the level of KIR2DL1 expression was positively correlated with the amount of MHC-I required for cytotoxicity inhibition (**P<0.005), such that NK cell clones expressing KIR2DL1 are inhibited by low amounts of target cell surface HLA-Cw6 (on average 2×10^4^ molecules) while KIR2DL1-negative cells are inhibited only by high MHC-I levels (Figure 3; see also Table S1 for a statistical analysis comparing different receptor combinations). No other receptors tested had a strong correlation with the EC50 (Figure 3C,D). Thus, we conclude that expression of KIR2DL1 determines the amount of target HLA-Cw6 required for NK cell inhibition, that is, the cell's activation/inhibition threshold.

### NK cell clones differ from each other mostly in their activation/inhibition thresholds

**Figure 4:**
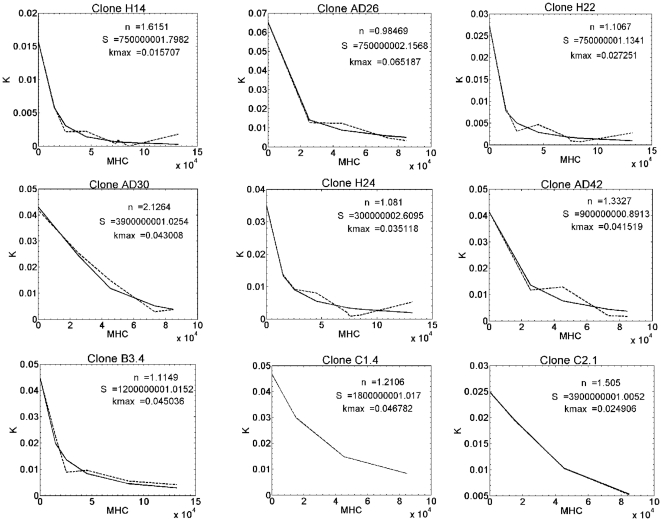
Sample fitting graphs. The dependence of target cell lysis on target cell MHC expression level. Dashed lines: *κ* values calculated from experimental lysis data based on Figure S1D. Solid lines: the best fit of model results to the data (in clones C1.4 and C2.1 these lines are masked by the dashed lines). The values of *n*, *S* and *κ_max_* that yielded best fit for each clone modeled are indicated on the figures. The parameter value ranges used in these runs were: *n* was 0.25–3.00, varied in 23 steps of 0.125; *S* was 0–5*10^9^, varied in 101 steps of 5*10^7^; *κ_max_* was 0.01–0.21, varied in 21 steps of 0.01.

In order to understand how KIR2DL1 expression levels determine NK cell activation/inhibition thresholds, we mathematically modelled the dependence of the killing rate *k* on the product of the numbers of KIR and MHC molecules by a sigmoid threshold function (equation 1), where *S* denotes the threshold of the NK cell clone in the same experiment, in units of (molecule numbers)^2^ as it is given in terms of the MHC*KIR product. The parameter *κ_max_* is the maximum killing probability of an NK cell belonging to each clone, per encounter, and *n* is the exponent of the sigmoid function. For each NK cell clone, we performed a curve-fit of equation 1 to the data on percent of target cells lysed as function of MHC expression levels (9 representative examples are given in Figure 4). The parameter value ranges used in these runs were: *n* was 0.25–3.00, varied in 23 intervals of 0.125; *S* was 0–5*10^9^, varied in 101 intervals of 5*10^7^; *κ_max_* was 0.01–0.21, varied in 21 intervals of 0.01.

Values of *κ_max_* obtained for different clones varied between 0.015 and 0.065 per cell per encounter, that is, about four-fold. Values of *n* (response sensitivity exponent) varied between 0.99 and 2.36, that is, only about two-fold. Moreover, all clones but one (AD30) had *n* ≤ 1.61, that is, most of the variation in *n* was of less than two-fold. In contrast, values of *S* (the threshold parameter) varied by more than ten-fold – from 3*10^8^ to 3.9*10^9^. These results show that, in addition to the cell surface expression levels of KIR2DL1, the clones differ mostly in their intrinsic activation thresholds (*S*), which may have been set during the education process.

### Target cell killing by NK cells may require NK cell priming

**Figure 5:**
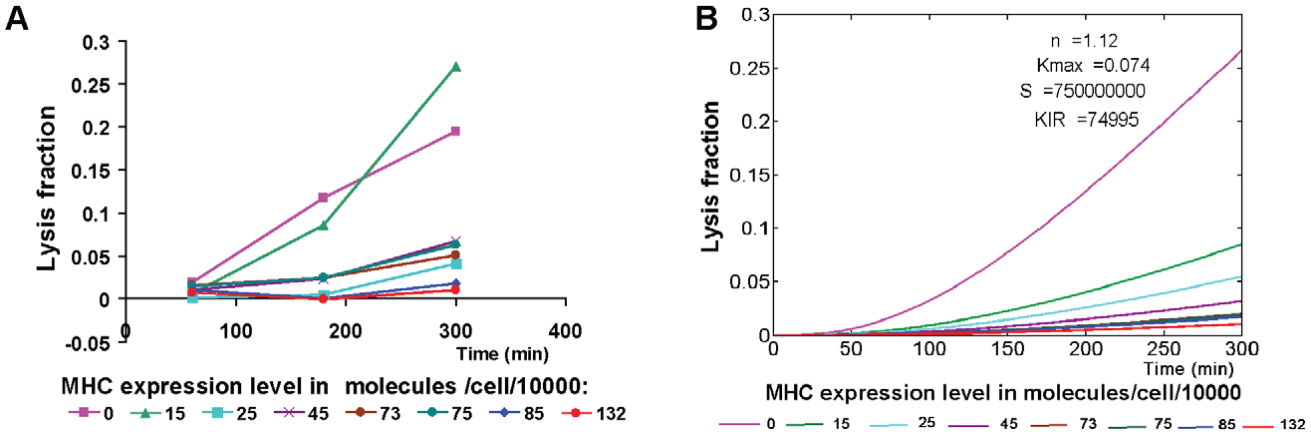
The kinetics of NK cell activation. A) Experimental results. The percent of lysed target cells expressing different amounts of MHC class I was determined by radioactive release assays, after 1, 3 or 5 hrs of co-incubation. B) Mathematical model – simulation results.

To explore the kinetics of NK cell activation and target cell lysis, we applied a combination of mathematical and experimental tools. With all target cell clones except the two with zero or lowest MHC expression, lysis (determined by radioactivity release assays) is hardly seen after 3 hours; only after 5 hours some lysis is seen in all target cells (Figure 5A). This contrasted with our observation (Figure S1) and others' that conjugation reaches saturation in the first half-hour of the experiment [20], and raised the question of what causes the delay in observation of target cell lysis. To address this issue, we simulated the kinetics of NK cell activation under three alternative mathematical models. The simple model, in which any target cell-NK cell encounter may result in immediate target cell killing (Figure 1A and equations 2–4), always resulted in convex graphs of lysis vs. time (data not shown), which did not resemble the concave graphs observed experimentally. Hence, we needed to include some delay mechanism in the model.

The delay in observation of lysis may be due to the fact that target cells take some time from the moment they receive the death signal until they fully die and disintegrate [30]. During this time, they would still be counted as present and alive in the culture. This feature was implemented in the second version of the model by including target cell death delay (Figure 1B and equations 2–5; the number of dying target cells is denoted by *T_d_*, and the *T_d_* subset death rate is denoted by *µ*, whose units are (min)^−1^). However, simulations of this model, with *µ* obtained by fitting to data on NK cell line-mediated lysis of targets which express no MHC ligands, did not result in concave graphs either; the behaviour of this model differed only slightly from that of the simple model (data not shown). Thus, target cell death delay alone could not explain the dynamics of lysis.

An alternative hypothesis was that NK cells require some priming before they can lyse the target cells [31], such that, if we start with resting NK cells, each cell becomes activated – and can lyse a target cell – only after its first encounter with a target cell. In the first encounter the NK cell will not kill the target cell. This “NK cell priming” hypothesis was implemented in the third version of the model, which includes two NK cell subsets, resting and activated (Figure 1C and equations 6–10). Simulations of this model yielded lysis kinetics which resembled the experimental kinetics much better than those of the earlier versions (Figure 5B). While the resemblance here is only qualitative due to data paucity (there was only one data point per MHC level per time point), it is important to note that we could get this type of kinetics only when the model included NK cell priming; assuming both subsets can lyse target cells, and differ only in their conjugation rate, dissociation rate, and/or *κ_max_*, always yielded graphs that were more similar to those of the simple model than to the experimentally observed ones (data not shown).

## Discussion

This paper addressed the question of how the activation/inhibition decision by an NK cell is regulated by the expression level of MHC class I on the target cell. More specifically, we asked what determines the NK cell's activation threshold, and what the functional implications of this threshold are.

We show here that human NK cells and NK cell clones have different stimulation requirements for degranulation and for IFN-γ secretion, with the latter having more stringent stimulation requirements and taking longer to be elicited. These data are consistent with observations in mice [6], [8], [32] and in polyclonal human NK cells stimulated with *Drosophila* cells transfected to express different combinations of ligands [24]. It seems important that NK cells are able to control IFN-γ secretion in a more stringent way than cytotoxicity, to avoid unnecessary stimulation of other cells while ensuring lysis of any dangerous cell. On the other hand, we show that while CD107a expression and IFN-γ secretion are not correlated, their degrees of inhibition by MHC-I molecules are correlated (Figure 2D). This implies that inhibitory receptor binding to MHC class I inhibits both responses in the same way.

Analyzing NK cell inhibition in more detail, we found that different NK cells differ mostly in the number of target MHC molecules required to inhibit their responses, that is, in their activation/inhibition threshold (Figure 4). The rheostat model by Höglund *et al.* [6], [17], [33], [34] proposes that NK cell education is not a binary selection of NK cells that do or do not express self MHC-specific receptors, but rather a quantitative tuning of each cell's activation threshold in proportion to inhibitory signals received during its education. The differences observed here between activation thresholds of different NK cell clones may indeed result from differences in the inhibitory signals these cells have received during education.

Consistent with this line of thought, we found that the expression level of KIR2DL1 determined the inhibition threshold of target HLA-Cw6 molecules on NK cells, with little contribution from other NK cell receptors (Figure 3 and Table S1). However, as experiments were performed with primary human NK clones possessing variegated receptor expression, we cannot completely exclude the possibility that the strong effect KIR2DL1 has in regulating the inhibitory threshold may mask a weaker effect of other NK receptors, as shown by, e.g., the small increase in R^2^ obtained for combinations of KIR2DL1 with LIR1 relative to R^2^ of KIR2DL1 alone. Nevertheless, the findings that KIR2DL1 and not other receptors is the main player determining HLA-Cw6-mediated thresholds on NK cells, and that human NK cell clones vary in their intrinsic activation thresholds, may be important for predicting the outcome of allogeneic haematopoietic stem cell transplantation in treatment of acute myeloid leukemia, where NK cell activation plays a major role [35].

NK cells not expressing KIR2DL1 could still be inhibited by MHC-I, though this required a higher number of MHC class I molecules than cells expressing KIR2DL1 (requiring approximately 5×10^4^ MHC class I molecules for inhibition as opposed to 2×10^4^, a range that includes the amount of MHC class I protein expressed by different primary and immortal cell lines [1]). Specific binding to NK cell receptors leads to MHC-I transfer to the NK cells. The percentage of NK cells acquiring target HLA-Cw6 molecules is almost null in contacts involving KIR2DL1^−^ cells [36], indicating that receptors other than KIR2DL1 govern this NK-target cell interaction. Inhibition of cytotoxicity on NK cells not expressing KIR2DL1 is likely to be mediated by HLA-E, since HLA-Cw6 codes for a leader peptide which can be presented by HLA-E. It will be interesting to determine whether expression of HLA-E correlates with expression levels of HLA-Cw6. It is however unlikely that, in the presence of KIR2DL1, the interaction of HLA-E with CD94/NKG2A has played a significant role in determining the cells' MHC-I-mediated threshold, because our statistical analysis has shown that the expression of KIR2DL1, and not CD94/NKG2A, correlated with NK cell inhibition. Thus, NK cell clones expressing KIR2DL1 will be inhibited by low amounts of HLA-Cw6, while cells not expressing KIR2DL1 will only be inhibited by high amounts of HLA-Cw6. These data add to the results obtained with precursor –B-cell leukemia target cells, where it was described that expression of KIR by NK cells affects their capacity to lyse target cells expressing high or low amounts of HLA class I [16].

The mechanism of the putative NK cell priming is yet unknown. Expression of FasL was observed to be upregulated upon NK cell activation by stimulating the cells with PMA/ionomycin, with cytokines, or by cross-linking different activating receptors (see, e.g. [37], [38]). Thus, FasL expression may be regulated by the presence of target cells. On the other hand, since the preferential mechanism for NK cytotoxicity is via perforin and granzymes [38], it would be interesting to check whether there are changes in this mechanism upon first interaction with target cells. Finally, the mechanism may not necessarily be direct; it could also be the result of interactions with DC rather than with target cells, or both mechanisms could play a role. In future studies we plan to directly test this priming hypothesis, and elucidate the responsible mechanisms.

## Supporting Information

Figure S1 **Parameter determination for the mathematical model.** (A) The blue points represent data of percentage of conjugated target cells out of the initial target cell number in conjugation assays, performed at a 1∶1 ratio, with peripheral blood polyclonal NK cells and with 221 target cells. The pink line represents the model's best fit to these data, which was obtained with β = 3.56×10−6 (cells*minutes)-1 and τ = 16.75 (minutes). For conjugation assays, NK cells and target cells were incubated for various times at a 1∶1 ratio (2.5×105 of cells for each) in 50 µl of RPMI plus supplements. After the incubation, cells were fixed in 300 µl of Cytofix/CytopermTM (BD Biosciences) for 15 min at 4°C. Cells were washed twice with PBS 1x/1% BSA/0.01% sodium azide and stained with a PE-Cy5 conjugated anti-human CD56 antibody and a FITC-conjugated anti-human CD19 antibody for 30 min at 4°C. Cells were washed twice with PBS 1x/1% BSA/0.01% sodium azide and analysed by flow cytometry. Isotype matched antibodies were used as controls. Data were analysed with CellQuest (Becton Dickinson). (B) cell fractions. Q –fraction of conjugated NK cells out of the initial number of NK cells (N0); N1 – fraction of free NK cells out of the initial number of NK cells (N0); M –fraction of free target cells out of the initial number of target cells (M0); fT – fraction of living target cells (free and conjugated) out of the total initial number of target cells (M0). Parameter values are as in Figure S1A. (C) Cell fractions are defined as in Figure S1B. Parameter values are as in Figure S1A, except that here κ = 0.05. (D) Lysed target cell fractions after 300 minutes of encountering NK cells for varying values of κ. Other parameter values are as in Figure S1A.
(DOC)

Figure S2 **Degranulation cannot always be a marker for lysis**, because it is not correlated with lysis for all clones. Examples of data for 5 clones are shown.
(DOC)

Figure S3 **Different NK cell clones express different receptor combinations.** The figure shows phenotypes of NK cell clones showing different lytic activity against target cells expressing different amounts of surface MHC class I. Each row refers to one clone. (**A**), (**B**) and (**C**) show data for clones were cytotoxicity decreased (A), did not change (B), or increased (C) with increasing expression of target cell MHC class I protein. Some clones did not efficiently lyse 221 target cells (**D**). The amount of MHC class I protein required to halve the maximum lysis (EC50) was classified into low, medium or high. Clones were screened for EB6 staining (KIR2DL/S1), LIR1, CD94, NKp46 and NKG2A expression, which were also classified into similar levels.
(DOC)

Table S1 **The NK cell inhibition threshold is mainly determined by KIR2DL1.** A regression analysis was performed for all the receptor combinations, to evaluate the contribution of each receptor – or combination of receptors – to the EC50. R^2^ is the correlation measure, and its range of values is from -1 to 1, with 1 indicating a full direct correlation. By looking on the effect of each receptor alone on the EC50, we see that KIR2DL1 has the strongest effect (R^2^ = 0.24). However, the combinations show that KIR2DL1 and NKp46 together affect the EC50 even more (R^2^ = **0.63**). Not all receptor combinations were observed in the experiments (marked as na, that is, not available), therefore there might be other combinations that have higher effects on the EC50 and the inhibition. The validity of some of the regression model fits was indicated by the software (SAS) as questionable (cases marked with asterisks), presumably because of the low sample sizes, as there were too few cases for some of the receptor combinations.
(DOC)
