# Supplementary material for: Human NK Cells Differ More in Their KIR2DL1-Dependent Thresholds for HLA-Cw6-Mediated Inhibition than in Their Maximal Killing Capacity
Source: PLoS One. 2011 Sep 19;6(9):e24927. doi: 10.1371/journal.pone.0024927 (PMC3176315; doi:10.1371/journal.pone.0024927)
Supplement: Figure S1 — Parameter determination for the mathematical model. (A) The blue points represent data of percentage of conjugated target cells out of the initial target cell number in conjugation assays, performed at a 1∶1 ratio, with peripheral blood polyclonal NK cells and with 221 target cells. The pink line represents the model's best fit to these data, which was obtained with β = 3.56×10^−6^ (cells*minutes)-1 and τ = 16.75 (minutes). For conjugation assays, NK cells and target cells were incubated for various times at a 1∶1 ratio (2.5×10^5^ of cells for each) in 50 µl of RPMI plus supplements. After the incubation, cells were fixed in 300 µl of Cytofix/CytopermTM (BD Biosciences) for 15 min at 4°C. Cells were washed twice with PBS 1x/1% BSA/0.01% sodium azide and stained with a PE-Cy5 conjugated anti-human CD56 antibody and a FITC-conjugated anti-human CD19 antibody for 30 min at 4°C. Cells were washed twice with PBS 1x/1% BSA/0.01% sodium azide and analysed by flow cytometry. Isotype matched antibodies were used as controls. Data were analysed with CellQuest (Becton Dickinson). (B) cell fractions. Q –fraction of conjugated NK cells out of the initial number of NK cells (N0); N1 – fraction of free NK cells out of the initial number of NK cells (N0); M –fraction of free target cells out of the initial number of target cells (M0); fT – fraction of living target cells (free and conjugated) out of the total initial number of target cells (M0). Parameter values are as in Figure S1A. (C) Cell fractions are defined as in Figure S1B. Parameter values are as in Figure S1A, except that here κ = 0.05. (D) Lysed target cell fractions after 300 minutes of encountering NK cells for varying values of κ. Other parameter values are as in Figure S1A. (DOC) [file pone.0024927.s001.doc]

**Figure S1: Parameter determination for the mathematical model**

(A) The blue points represent data of percentage of conjugated target cells out of the initial target cell number in conjugation assays, performed at a 1:1 ratio, with peripheral blood polyclonal NK cells and with 221 target cells. The pink line represents the model’s best fit to these data, which was obtained with β=3.56x10^–6^ (cells*minutes)^-1^ and τ=16.75 (minutes). For conjugation assays, NK cells and target cells were incubated for various times at a 1:1 ratio (2.5×10^5^ of cells for each) in 50μl of RPMI plus supplements. After the incubation, cells were fixed in 300µl of Cytofix/Cytoperm^TM^ (BD Biosciences) for 15min at 4ºC. Cells were washed twice with PBS 1x/1% BSA/0.01% sodium azide and stained with a PE-Cy5 conjugated anti-human CD56 antibody and a FITC-conjugated anti-human CD19 antibody for 30min at 4ºC. Cells were washed twice with PBS 1x/1% BSA/0.01% sodium azide and analysed by flow cytometry. Isotype matched antibodies were used as controls. Data were analysed with CellQuest (Becton Dickinson). (B) cell fractions. Q – fraction of conjugated NK cells out of the initial number of NK cells (N_0_); N1 – fraction of free NK cells out of the initial number of NK cells (N_0_); M –fraction of free target cells out of the initial number of target cells (M_0_); fT – fraction of living target cells (free and conjugated) out of the total initial number of target cells (M_0_). Parameter values are as in Figure S1A. (C) Cell fractions are defined as in Figure S1B. Parameter values are as in Figure S1A, except that here κ=0.05. (D) Lysed target cell fractions after 300 minutes of encountering NK cells for varying values of κ. Other parameter values are as in Figure S1A.


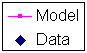


A)


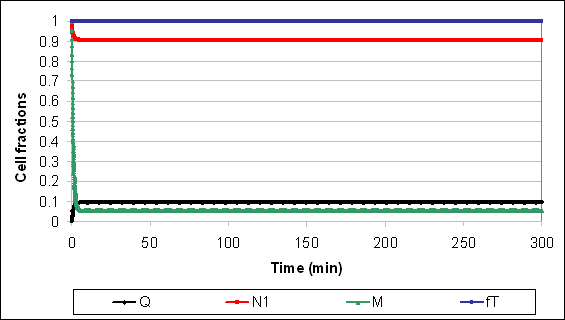
B)


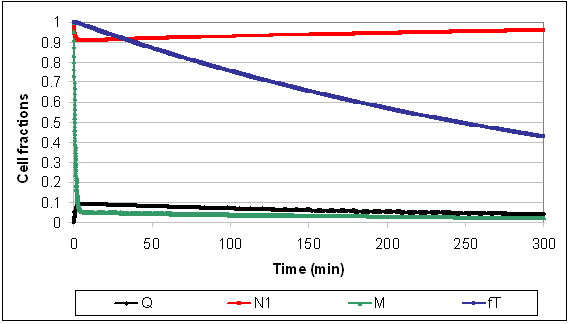
C)

D)

**Lysis fraction**
