# Supplementary material for: Human NK Cells Differ More in Their KIR2DL1-Dependent Thresholds for HLA-Cw6-Mediated Inhibition than in Their Maximal Killing Capacity
Source: PLoS One. 2011 Sep 19;6(9):e24927. doi: 10.1371/journal.pone.0024927 (PMC3176315; doi:10.1371/journal.pone.0024927)
Supplement: Figure S2 — Degranulation cannot always be a marker for lysis, because it is not correlated with lysis for all clones. Examples of data for 5 clones are shown. (DOC) [file pone.0024927.s002.doc]

**Figure S2:** Degranulation cannot always be a marker for lysis, because it is not correlated with lysis for all clones. Examples of data for 5 clones are shown.
