# Supplementary material for: Human NK Cells Differ More in Their KIR2DL1-Dependent Thresholds for HLA-Cw6-Mediated Inhibition than in Their Maximal Killing Capacity
Source: PLoS One. 2011 Sep 19;6(9):e24927. doi: 10.1371/journal.pone.0024927 (PMC3176315; doi:10.1371/journal.pone.0024927)
Supplement: Figure S3 — Different NK cell clones express different receptor combinations. The figure shows phenotypes of NK cell clones showing different lytic activity against target cells expressing different amounts of surface MHC class I. Each row refers to one clone. (A), (B) and (C) show data for clones were cytotoxicity decreased (A), did not change (B), or increased (C) with increasing expression of target cell MHC class I protein. Some clones did not efficiently lyse 221 target cells (D). The amount of MHC class I protein required to halve the maximum lysis (EC50) was classified into low, medium or high. Clones were screened for EB6 staining (KIR2DL/S1), LIR1, CD94, NKp46 and NKG2A expression, which were also classified into similar levels. (DOC) [file pone.0024927.s003.doc]

**Figure S3:** **Different NK cell clones express different receptor combinations.** The figure shows phenotypes of NK cell clones showing different lytic activity against target cells expressing different amounts of surface MHC class I. Each row refers to one clone. (**A**), (**B**) and (**C**) show data for clones were cytotoxicity decreased (A), did not change (B), or increased (C) with increasing expression of target cell MHC class I protein. Some clones did not efficiently lyse 221 target cells (**D**). The amount of MHC class I protein required to halve the maximum lysis (EC50) was classified into low, medium or high. Clones were screened for EB6 staining (KIR2DL/S1), LIR1, CD94, NKp46 and NKG2A expression, which were also classified into similar levels.

**
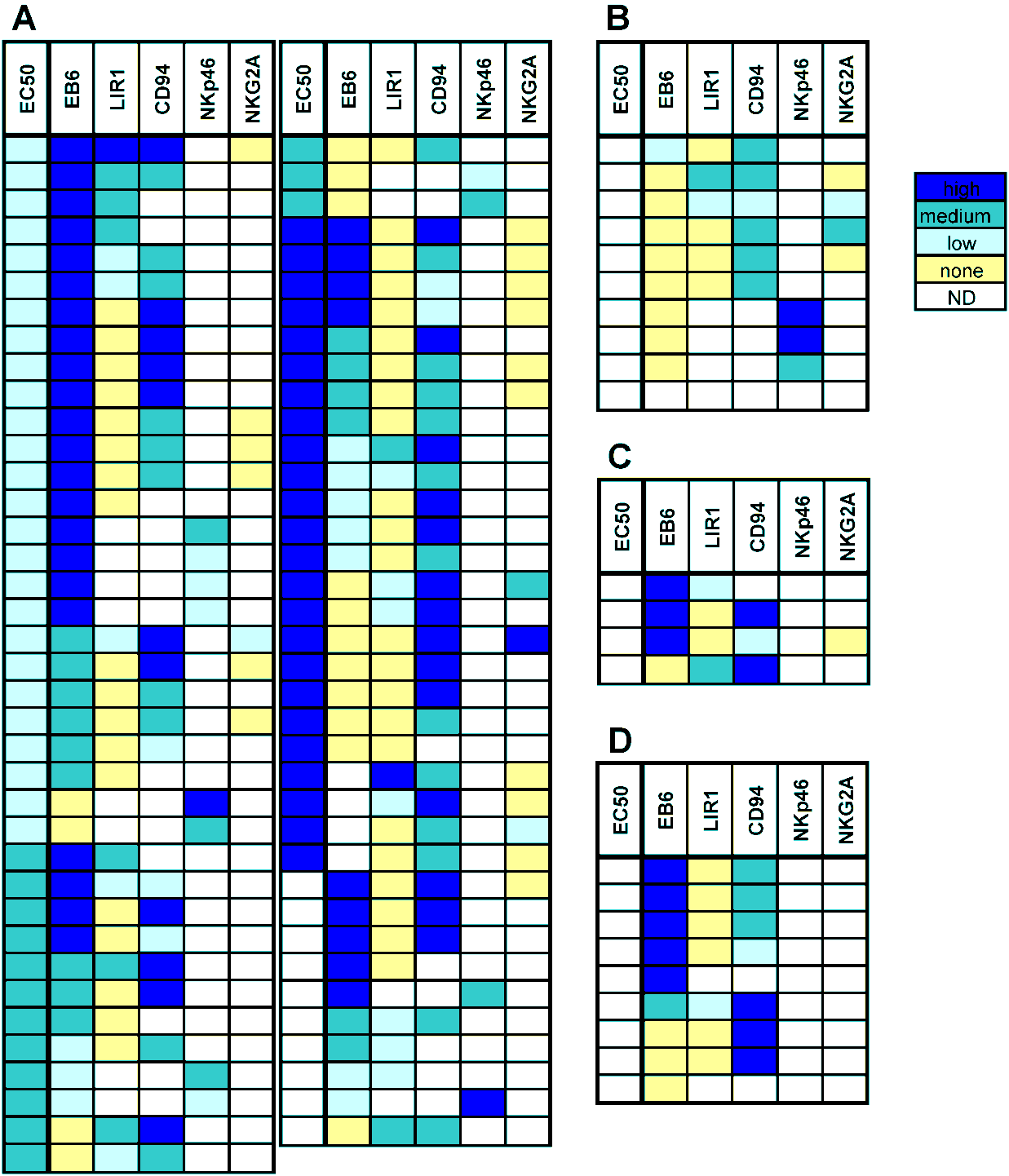
**
