# Supplementary material for: Human NK Cells Differ More in Their KIR2DL1-Dependent Thresholds for HLA-Cw6-Mediated Inhibition than in Their Maximal Killing Capacity
Source: PLoS One. 2011 Sep 19;6(9):e24927. doi: 10.1371/journal.pone.0024927 (PMC3176315; doi:10.1371/journal.pone.0024927)
Supplement: Table S1 — The NK cell inhibition threshold is mainly determined by KIR2DL1. A regression analysis was performed for all the receptor combinations, to evaluate the contribution of each receptor – or combination of receptors – to the EC50. R2 is the correlation measure, and its range of values is from -1 to 1, with 1 indicating a full direct correlation. By looking on the effect of each receptor alone on the EC50, we see that KIR2DL1 has the strongest effect (R2 = 0.24). However, the combinations show that KIR2DL1 and NKp46 together affect the EC50 even more (R2 = 0.63). Not all receptor combinations were observed in the experiments (marked as na, that is, not available), therefore there might be other combinations that have higher effects on the EC50 and the inhibition. The validity of some of the regression model fits was indicated by the software (SAS) as questionable (cases marked with asterisks), presumably because of the low sample sizes, as there were too few cases for some of the receptor combinations. (DOC) [file pone.0024927.s004.doc]

**Table S1**: The NK cell inhibition threshold is mainly determined by KIR2DL1.

| **Receptor combination** | **R^2^ for EC50** |
| --- | --- |
| **KIR2DL1** | **0.24** |
| **LIR1** | 0.02 |
| **CD94** | 0.00 |
| **NKp46** | 0.02 |
| **NKG2A** | 0.07 |
| **KIR2DL1 + LIR1** | 0.35 |
| **KIR2DL1 + CD94** | 0.29 |
| **KIR2DL1 + NKp46** | **0.63*** |
| **KIR2DL1 + NKG2A** | 0.12 |
| **LIR1 + CD94** | 0.01 |
| **LIR1 + NKp46** | na |
| **LIR1 + NKG2A** | 0.08 |
| **CD94 + NKp46** | na |
| **CD94 + NKG2A** | 0.20 |
| **KIR2DL1 + LIR1+ CD94** | 0.32 |
| **KIR2DL1 + LIR1+ NKp46** | na |
| **KIR2DL1 + LIR1+ NKG2A** | 0.42 |
| **KIR2DL1 + CD94+ NKp46** | na |
| **KIR2DL1 + CD94+ NKG2A** | 0.35* |
| **LIR1 + CD94 + NKp46** | na |
| **LIR1 + CD94 + NKG2A** | 0.21 |
| **KIR2DL1 + LIR1+ CD94 +NKp46** | na |
| **KIR2DL1 + LIR1+ CD94 +NKG2A** | 0.48* |
| **LIR1+ CD94 +NKp46 +NKG2A** | na |
| **KIR2DL1 + LIR1+ CD94 + NKp46 + NKG2A** | na |

A regression analysis was performed for all the receptor combinations, to evaluate the contribution of each receptor – or combination of receptors – to the EC50. R^2^ is the correlation measure, and its range of values is from -1 to 1, with 1 indicating a full direct correlation. By looking on the effect of each receptor alone on the EC50, we see that KIR2DL1 has the strongest effect (R^2^=0.24). However, the combinations show that KIR2DL1 and NKp46 together affect the EC50 even more (R^2^=**0.63).** Not all receptor combinations were observed in the experiments (marked as na, that is, not available), therefore there might be other combinations that have higher effects on the EC50 and the inhibition. The validity of some of the regression model fits was indicated by the software (SAS) as questionable (cases marked with asterisks), presumably because of the low sample sizes, as there were too few cases for some of the receptor combinations.
